# A novel biosensor based on intestinal 3D organoids for detecting the function of BCRP

**DOI:** 10.1080/10717544.2017.1381199

**Published:** 2017-09-26

**Authors:** Lei Zhang, Junfang Zhao, Chenmeizi Liang, Mingyao Liu, Feng Xu, Xin Wang

**Affiliations:** a East China Normal University and Shanghai Fengxian District Central Hospital Joint Research Center for Translational Medicine, Shanghai Key Laboratory of Regulatory Biology, Institute of Biomedical Sciences and School of Life Sciences, East China Normal University, Shanghai, China;; b Department of Pharmacy, Shanghai Fengxian District Central Hospital, Shanghai, China;; c Department of Molecular and Cellular Medicine, Institute of Biosciences and Technology, Texas A&M University Health Science Center, Houston, TX, USA

**Keywords:** BCRP, transporter, Hoechst 33342, 3D organoids, small intestine

## Abstract

Breast cancer resistance protein (BCRP), a key drug efflux transporter, significantly affects the therapeutic efficacy of many drugs. Thus, screening specific BCRP inhibitors and distinguishing between substrates and non-substrates of BCRP are valuable in drug discovery and development. This study presents a novel BCRP biosensor based on intestinal 3D organoids for rapid and sensitive detection of BCRP function. First, the crypts were isolated from mouse small intestine, and cultured in advanced DMEM/F12 medium to develop intestinal 3D organoids. Second, immunohistochemical studies demonstrated that BCRP protein in the organoids presented a similar expression and physiologic position to the small intestinal epithelium. Finally, the cultured organoids were treated in BCRP fluorogenic probe substrate Hoechst 33342 with or without BCRP inhibitor Ko143 and YHO-13177. The fluorescence intensity of Hoechst 33342 released from inner of the organoids was detected by microplate reader and the concentrations were calculated. Ko143 and YHO-13177 significantly inhibited the BCRP-mediated Hoechst 33342 transport in the 3D organoids. Consequently, a rapid and efficient biosensor has been successfully established to study BCRP, especially screening BCRP inhibitors in a high-throughput way.

## Introduction

The ATP-binding cassette (ABC) transporters participate in the transport of a wide large number of endogenous and exogenous substrates, such as cholesterol, bile salt, peptides, nucleosides, drugs, chloride ion, toxins, organic anions, iron, and sterols across extra- and intracellular membranes (Glavinas et al., 2004; Hegedus et al., 2012). In all ABC transporters, the breast cancer resistance protein (BCRP, ABCG2), P-glycoprotein (P-gp, ABCB1), and the MDR-associated protein-1 (MRP1, ABCC1) are associated with multidrug resistance (Choudhuri & Klaassen, 2006; Robey et al., 2009). In particular, drug resistance is a principal problem in cancer chemotherapy. The most important mechanism of drug resistance is that ABC transporters pump a variety of structurally unrelated anticancer drugs, such as the taxanes, vinca alkaloids and anthracycline from the cancer cell, thus leading to reduce intracellular accumulation of the drugs (Gottesman et al., 2002; Westover & Li, 2015).

Breast cancer resistance protein is an important multidrug resistance protein and significantly affects the treatment results of many drugs, especially anticancer drugs (Doyle & Ross, 2003). Hence, identifying substrates, non-substrates or inhibitors of BCRP is vital not only for clinical use but also for drug discovery and development. BCRP has been found to efflux mitoxantrone, methotrexate, gefitinib, imatinib and camptothecin including 7-ethyl-10-hydroxycamptothecin (SN-38, irinotecan active metabolites), thus reducing intracellular drug concentration and the therapeutic effect (Yamazaki et al., 2011). In addition, BCRP in the intestine has been regarded as a major determinant of the bioavailability and clinical outcome of orally administered BCRP substrates such as topotecan (Kruijtzer et al., 2002). Furthermore, BCRP is highly expressed in cancer stem cells (Haraguchi et al., 2006). In fact, inhibition of drug efflux will be beneficial to reverse BCRP-mediated drug resistance via using small molecule compounds such as fumigatus C (Rabindran et al., 1998), GF120918 (Jonker et al., 2000), neonomycin (Shiozawa et al., 2004), and genistein (Imai et al., 2004). Currently, however, no inhibitor specifically targeting BCRP has been used in clinic (Yamazaki et al., 2011). Therefore, more specific BCRP inhibitors that could be clinically useful drugs are needed to develop for conquering drug resistance.

The major methods are used to identify the interaction of small molecules with BCRP *in vitro* including the model of BCRP-overexpressing cell lines and monolayers with Caco-2 cells. For example, an *in vitro* method based on the Madin–Darbin canine kidney cells overexpressing mouse BCRP was established to verify the substrates/inhibitors of BCRP (Muenster et al., 2008). However, the above method is generally consuming time and expensive due to construction of overexpression vectors and screening stable overexpressing cell lines. In addition, monolayers with Caco-2 cells are the most widely used in the pharmaceutical industry for studying BCRP-mediated compounds cross-cell transport (Liang et al., 2000). But, there are still many disadvantages including the tighter cellular junctions than normal enterocytes and the long culture period of at least 21 days (Zhao et al., 2017). Therefore, it is urgent to establish a new model for the study of BCRP-mediated drug transport *in vitro*.

Herein, we reported a novel biosensor based on intestinal three-dimensional (3D) organoids for the study of BCRP, especially rapid and sensitive detection of BCRP inhibitors using BCRP fluorescent substrate Hoechst 33342. In this assay, the mouse small intestinal crypts were firstly isolated and cultured to develop 3D organoids. BCRP expression in cultured organoids were assessed by both mRNA and immunohistochemical analysis at the protein level. Hoechst 33342 as a BCRP fluorogenic probe substrate could be detected sensitively by monitoring the change of fluorescence signal. Furthermore, the effects of BCRP inhibitor Ko143 and YHO-13177 on accumulation of Hoechst 33342 in the cultured organoids were analyzed to verify the feasibility and validity of the model.

## Experimental

### Chemicals and reagents

Hoechst 33342, Ko143 and YHO-13177 were purchased from Med Chem Express (Monmouth Junction, NJ, USA). Recombinant human R-spondin 1 and recombinant mouse noggin were obtained from Pepro Tech Inc. (Rocky Hill, NJ, USA). Mouse recombinant EGF, advanced DMEM/F12, B-27 supplement and N-2 supplement were supplied by Invitrogen (Carlsbad, CA, USA). Matrigel (GFR, phenol-free) was bought from Corning Inc. (Bedford, MA, USA). Anti-BCRP antibody and goat anti-rabbit antibody were purchased from Abcam (Cambridge, UK). Trizol, PrimeScript™ RT reagent Kit and SYBR^®^ Premix Ex Taq™ II MIX were supplied by Takara (Dalian, China).

### Animals

C57BL/6 mice were purchased from National Rodent Laboratory Animal Resources (Shanghai, China). The animals were kept in a specific pathogen-free facility with access to rodent chow cubes and sterile water, with 12 h light-dark cycles. All the methods performed in animals were carried out in accordance with the National Institutes of Health standards established in the ‘Guidelines for the Care and Use of Experimental Animals’. All the experiments in animals were approved by the Ethics Committee on Animal Experimentation of East China Normal University (Shanghai, China).

### Crypts isolation and organoids culture

Small intestinal crypts were isolated from eight-week old C57BL/6 mice and cultured into organoids as previously described (Zhang et al., 2016b; Riemer et al., 2017; Son et al., 2017). Briefly, crypts were released from small intestines by incubating in 2 mM EDTA in PBS for 30 min at 4 °C. The crypts were embedded in Matrigel and cultured in Advanced DMEM/F12 medium containing 500 ng/mL R-spondlin1, 50 ng/mL EGF and 100 ng/mL Noggin. The medium was changed every 2 days until the following use of cultured organoids.

### Total RNA isolation and quantitative real-time PCR

Total RNA was isolated from crypts, villus and organoids using Trizol reagent and the cDNA was synthesized using PrimeScript™ RT reagent Kit as previously described (Zhang et al., 2016a). Quantitative real-time PCR was performed using SYBR^®^ Premix Ex Taq™ II MIX in QuantStudio 5 Real-Time PCR Systems (Thermo Fisher Scientific, Waltham, MA, USA). Relative mRNA expression was determined by the ΔΔ*Ct* method and normalized to β-actin. The primer sequences were as follows: forward: 5′-GAACTCCAGAGCCGTTAGGAC-3′ and reverse: 5′-CAGAATAGCATTAAGGCCAGGTT-3′ for BCRP, forward: 5′-CACTGTCGAGTCGCGTCCA-3′ and reverse: 5′-TGA-CCCATTCCCACCATCAC-3′ for β-actin.

### Immunohistochemistry

On the fourth cultured day, the organoids were separated from the Matrigel. The organoids and mouse small intestinal tissue were fixed in 4% paraformaldehyde, dehydrated with conventional gradient ethanol, embedded in paraffin and serially sliced into 5-μm thickness. All sections were deparaffinized. Antigens were retrieved in citrate buffer, and blocked using 5% normal goat serum. The sections were incubated with anti-BCRP antibody (1:500 dilution) at 4 °C overnight, rinsed and incubated with anti-rabbit antibody (1:1000 dilution) at room temperature for 1 h, and detected by diaminobenzidine. The slides were then counterstained with hematoxylin, mounted with a coverslip, and images were acquired using Leica DM4000 microscope (Wetzlar, Germany).

### Hoechst 33342 transport assay in the 3D organoids

On the fourth cultured day, Matrigel was broken up by pipetting back and forth several times with 1 mL tips. Transfer the suspension into a 1.5-mL centrifuge tube on ice to thaw the Matrigel. The organoids were separated by centrifuging 5 min at 150*g*, 4 °C, and resuspended with medium and seeded in 96-well plates. The organoids were counted and incubated in 2.5 μM Hoechst 33342 with or without BCRP inhibitor YHO-13177 (20 μM) or Ko143 (15 μM) at 37 °C for 20, 40, 60, 80 and 100 min, respectively (Yamazaki et al., 2011; Caetano-Pinto et al., 2016). The organoids were transferred into 1.5-mL centrifuges, and washed with ice-cold PBS for three times. To release Hoechst 33342, the organoids were further incubated in pre-warmed PBS at 37 °C for 4 h. The suspensions were collected and detected fluorescence intensity of Hoechst 33342 using FLUOStar OPTIMA (Ortenberg, Germany) under the condition of *λ*_ex_*/λ*_em_ = 350 nm/460 nm.

### Statistical analysis

All data were presented as mean ± SEM. Statistical analysis between different groups was performed using two-tailed *t*-test done with Origin Pro 9.0, and *p* values less than .05 were considered to indicate statistical significance.

## Results and discussion

### Establishment of small intestinal 3D organoids culture system

**Figure 1.**
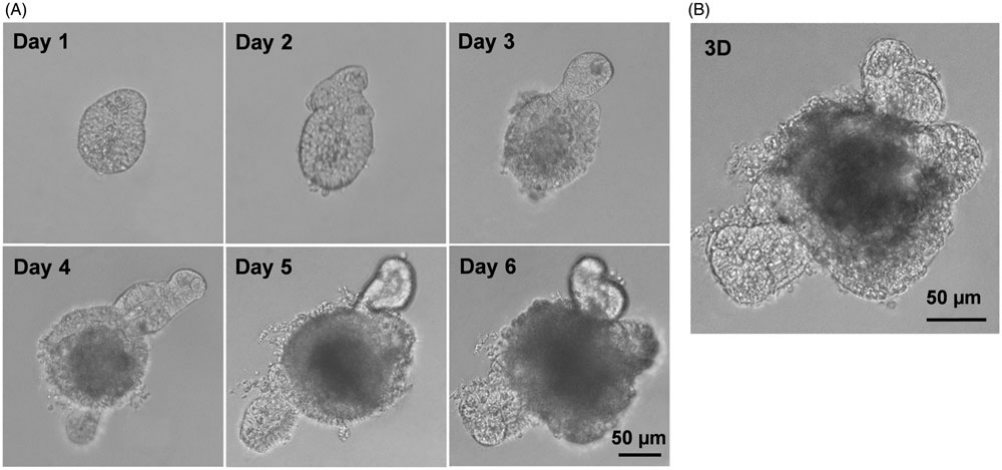
Establishment of small intestinal organoids culture system. Small intestinal crypts were isolated from C57BL/6 mouse small intestine, and were induced with cytokines to develop small intestinal organoids. (A) Time course of an isolated single crypt growth. (B) 3D reconstruction from these serial images. Scale bar: 50 µm.

The crypts were isolated from mouse small intestine, suspended in Matrigel, and induced with R-spondlin1, EGF and Noggin to develop small intestinal organoids. As shown in Figure 1, the crypts upper opening rapidly became sealed to form lumen, and subsequently underwent continuous budding events and further expansion to create organoids. These results were similar with previously described (Sato et al., 2009). Compared with our previous studies (Zhang et al., 2016b; Zhao et al., 2017), this study also presented that the special 3D structures of the organoids with an airtight cavity are suited for establishing a model of drug transport *in vitro*.

### The mRNA levels of BCRP in 3D organoids from different segments of small intestine

**Figure 2.**
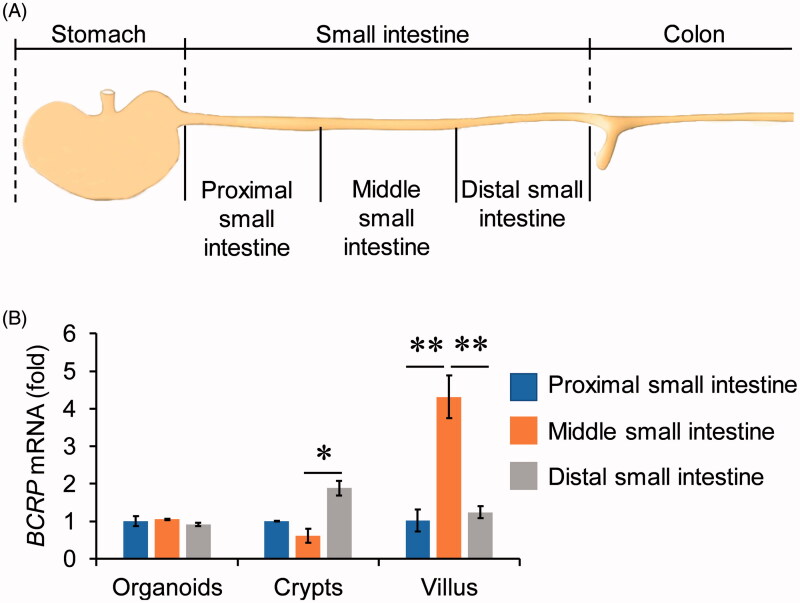
The expression of BCRP mRNA in organoids from different segments of small intestine. (A) Schematic of approaches to divide the small intestine into three segments. The small intestinal crypts and villus were isolated from the three segments, and then organoids were cultured from the three parts of crypts, respectively. (B) The mRNA levels of BCRP in the cultured organoids, crypts and villus from proximal, middle and distal small intestine. All data were presented as mean ± SEM (*n* = 3), *p ≤ .05 and **p ≤ .01.

To explore whether organoids cultured from different parts of small intestine had distinct patterns of BCRP expression profiling, the small intestine tissue was divided into proximal, middle, and distal small intestine, three equal parts along the proximal-to-distal axis (Figure 2(A)). The crypts and villus were isolated from the three segments, and then organoids were cultured from the three parts of crypts, respectively. Our data showed that the mRNA levels of BCRP in villus and crypts were different among proximal, middle, and distal small intestine (Figure 2(B)). Interestingly, however, the mRNA expression of BCRP was not distinct in the organoids cultured from three parts of small intestinal crypts (Figure 2(B)).

Although the mRNA levels of BCRP in middle small intestinal villus were obviously higher than proximal and distal small intestinal villus, the event did not occurred in the organoids cultured from the three parts of small intestinal crypts. A possible explanation of the phenomenon is that the crypt stem cells have distinct microenvironment and cytokines in different segments of small intestine. It should be noted that the numbers of crypts were gradually diminished from proximal to distal small intestine (data not shown). As a result, culturing organoids should be better to choose the parts from proximal to middle small intestine.

### The analogies of BCRP expression profiling between small intestinal villus and organoids

**Figure 3.**
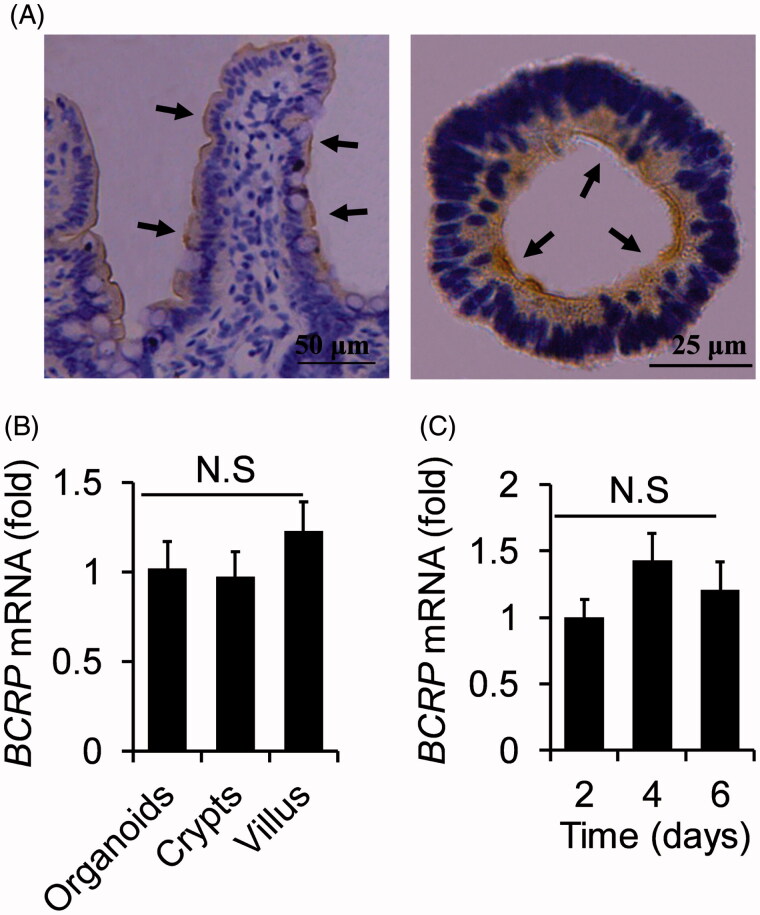
The analogies of BCRP expression profiling between small intestinal villus and organoids. (A) Location of BCRP protein was detected by immunohistochemistry in small intestinal villus and the cultured organoids, and were indicted by arrowheads. (B) The mRNA levels of *BCRP* in organoids at the fourth cultured day, villus and crypts from small intestine. (C) The mRNA expression of BCRP in the organoids on the different days of culture. All data were presented as mean ± SEM, *n* = 3 per group.

To further confirm that the organoids were suited for the study of BCRP-mediated drug transport, the protein expression of BCRP was also detected by immunohistochemistry with BCRP antibody in the both organoids and small intestine tissue. As shown in Figure 3(A), BCRP protein was expressed in the inner surface of organoids, while it was in the outside surface of small intestinal villus. Furthermore, the mRNA levels of *BCRP* were similar in organoids, villus and crypts from small intestine (Figure 3(B)). These results indicated that BCRP not only maintained physiological expression, but also remained at correct location in cultured organoids compared with small intestine tissue.

Moreover, we also analyzed the mRNA expression of BCRP in the organoids at different cultured days. The data presented that the mRNA levels of BCRP in the organoids were equivalent at different cultured two, four, and six days (Figure 3(C)). These results suggested the mRNA expression of BCRP was not influenced by the cultured time.

### BCRP-mediated Hoechst 33342 transport in the 3D organoids

**Figure 4.**
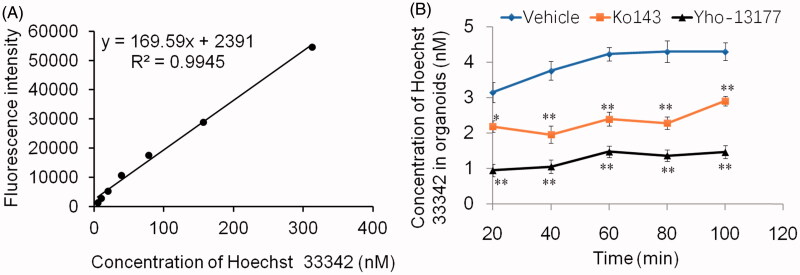
BCRP-mediated Hoechst 33342 transport in organoids. Fluorescence intensity of Hoechst 33342 were detected by Fluorescence microplate reader under the condition of *λ*_ex_*/λ*_em_ = 350 nm/460 nm. (A) Good liner relationship was obtained over the range of 4.9–312.5 nM for Hoechst 33342 in PBS. (B) The concentration of Hoechst 33342 released from inner of the cultured organoids at different time-points. All data were presented as mean ± SEM, *n* = 3 per group. *p ≤ .05 and **p ≤ .01 when compared to vehicle.

**Figure 5.**
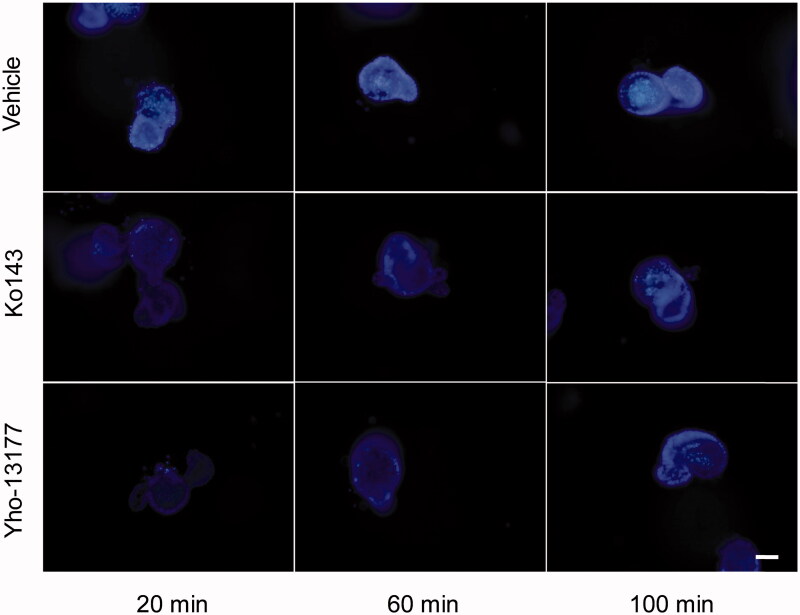
Fluorescent substrates accumulate in the organoids. The organoids were incubated in Hoechst 33342 with or without YHO-13177 or Ko143 for 20, 60 and 100 minutes, respectively. Ko143 and YHO-13177 notably decreased the fluorescence intensity of Hoechst 33342 in the organoids. Scale bar: 50 µm.

In order to verify the function of BCRP in the organoids, the BCRP-mediated Hoechst 33342 transport in the 3D organoids were carried out. Hoechst33342, a BCRP fluorogenic probe substrate, was chosen in this study. The fluorescence intensity of Hoechst 33342 released from inner of the organoids was detected using FLUOStar OPTIMA under the condition of *λ*_ex_*/λ*_em_ = 350 nm/460 nm. The detection assay displayed a good linearity of Hoechst33342 in range from 4.9 nM to 312.5 nM in PBS (*R*^2^ = 0.9945, Figure 4(A)). Based on this quantitative analysis method, the concentrations of Hoechst 33342 released from inner of the organoids were calculated. The data showed that BCRP mediated Hoechst 33342 transport into the lumen of organoids. At the same time, the fluorescence intensity of Hoechst 33342 in both control (no inhibitor) and BCRP inhibitor groups at different time-points was also investigated. As shown in Figure 4(B), the BCRP inhibitor Ko143 and YHO-13177 significantly reduced the accumulation of Hoechst 33342 in the organoids, suggesting inhibitory effects on the function of BCRP. Moreover, the accumulation of substrate in the organoids upon efflux inhibition was confirmed via fluorescence microscopy. Correspondingly, Ko143 and YHO-13177 significantly decreased the fluorescence intensity of Hoechst 33342 in the 3D organoids (Figure 5).

**Figure 6.**
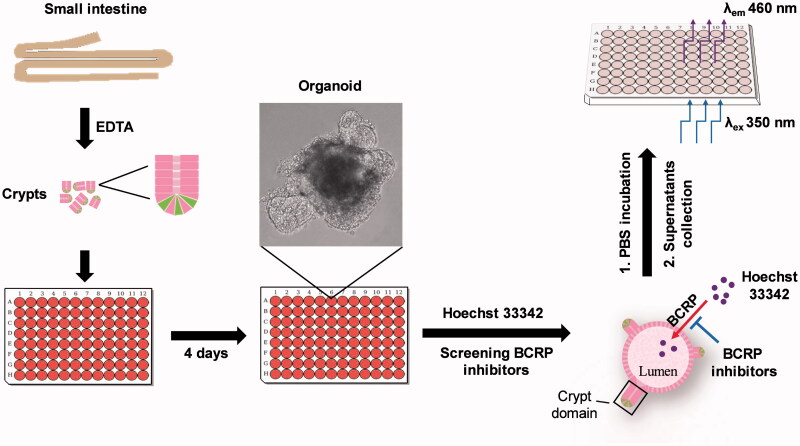
Schematic of approaches to screen inhibitors of BCRP using Hoechst 33342 transport in intestinal 3 D organoids. The crypts were isolated from mouse small intestine, and cultured to develop 3 D organoids. The cultured organoids were treated in BCRP fluorogenic probe substrate Hoechst 33342 with or without interesting compounds. The fluorescence intensity of Hoechst 33342 released from inner of the organoids was detected by microplate reader and the concentrations were calculated to screen inhibitors of BCRP.

To our knowledge, it is the first time to use intestinal 3D organoids to study BCRP-mediated drug transport via the measurement of Hoechst 33342. The scheme of the preparation of BCRP biosensor based on intestinal 3D organoids for detection of inhibitors of BCRP was summarized (Figure 6). Compared with previous models, there are following advantages of this BCRP fluorescent substrate biosensor. Firstly, the cultured organoids are directly differentiated from the primary stem cells of small intestine tissue, which are more similar to the native small intestinal epithelium. In particular, it takes a shorter culture period than the time of Caco-2 monolayer cell model (Aspenstrom-Fagerlund et al., 2012; Mease et al., 2012; Rodriguez-Colman et al., 2017). Secondly, the experimental assays based on MDCK-BCRP and LLC-PK1-BCRP overexpressing BCRP cell lines, are generally time consuming and expensive because of construction of overexpression vectors and screening stable overexpressing cell lines. In contrast, the model of the 3D organoids is established more easily. Finally, Hoechst 33342 in the cultured organoids are released by PBS incubation and are detected by fluorescence microplate reader, implying a rapid and efficient method for detection BCRP-mediated drug transport. The present BCRP biosensor not only demonstrated the function of BCRP in the 3D organoids, but also could be used to screen BCRP inhibitors.

Breast cancer resistance protein plays an important role in multidrug resistance, and significantly affects the therapeutic effects of many drugs. Thus, screening the specific inhibitor of BCRP is very significant. The present study pointed out a new way for the study of the inhibition of compounds on BCRP via calculation the accumulation of Hoechst 33342 in the intestinal 3D organoids. In addition, the present biosensor plus BCRP inhibitors as positive control, such as Ko143 and YHO-13177, could be used as a BCRP transport *in vitro* model for investigating the mechanism of drug resistance.

## Conclusions

In this study, a novel biosensor based on intestinal 3D organoids was successfully developed for the study of BCRP. The intestinal organoids were cultured from proximal to middle small intestine and developed 3D structures to form an airtight cavity, which provided a physical structure for drug transport. Moreover, BCRP protein expression in the organoids was similar to the small intestinal epithelium. By monitoring the change of fluorescence signal, Hoechst 33342 as a BCRP fluorogenic probe was detected sensitively in supernatant that released from the 3D organoids. Therefore, the present biosensor could be a promising tool to study BCRP, especially screening BCRP inhibitors in a high-throughput way.
